# Did a Complex Carbon Cycle Operate in the Inner Solar System?

**DOI:** 10.3390/life10090206

**Published:** 2020-09-16

**Authors:** Joseph A. Nuth, Frank T. Ferguson, Hugh G. M. Hill, Natasha M. Johnson

**Affiliations:** 1 Solar System Exploration Division, Code 690, NASA Goddard Space Flight Center, Greenbelt, MD 20771, USA; 2 Astrochemistry Laboratory, Code 691, NASA Goddard Space Flight Center, Greenbelt, MD 20771, USA; frank.ferguson@nasa.gov (F.T.F.); natasha.m.johnson@nasa.gov (N.M.J.); 3 Chemistry Department, Catholic University of America, 620 Michigan Ave., Washington, DC 20064, USA; 4 Physical Sciences, International Space University, 1 rue Jean-Dominique Cassini, 67400 Illkirch-Graffenstafden, France; hugh.hill@isunet.edu

**Keywords:** carbon depletion, solar nebula, surface-mediated reactions, planetesimal accretion

## Abstract

Solids in the interstellar medium consist of an intimate mixture of silicate and carbonaceous grains. Because 99% of silicates in meteorites were reprocessed at high temperatures in the inner regions of the Solar Nebula, we propose that similar levels of heating of carbonaceous materials in the oxygen-rich Solar Nebula would have converted nearly all carbon in dust and grain coatings to CO. We discuss catalytic experiments on a variety of grain surfaces that not only produce gas phase species such as CH_4_, C_2_H_6_, C_6_H_6_, C_6_H_5_OH, or CH_3_CN, but also produce carbonaceous solids and fibers that would be much more readily incorporated into growing planetesimals. CH_4_ and other more volatile products of these surface-mediated reactions were likely transported outwards along with chondrule fragments and small Calcium Aluminum-rich Inclusions (CAIs) to enhance the organic content in the outer regions of the nebula where comets formed. Carbonaceous fibers formed on the surfaces of refractory oxides may have significantly improved the aggregation efficiency of chondrules and CAIs. Carbonaceous fibers incorporated into chondritic parent bodies might have served as the carbon source for the generation of more complex organic species during thermal or hydrous metamorphic processes on the evolving asteroid.

## 1. Introduction

Dust in the interstellar medium (ISM) is a well-mixed distribution of carbonaceous and silicate grains [1,2] that ultimately serves as the source for the solids in the solar nebula. According to this model [1], roughly 88% of the carbon and 100% of silicon, iron, magnesium, and other metal oxides are in solids with a grain size distribution proportional to ~r^−3.5^. Graphite grains range from 0.005 to 1 micron in radius, while the silicate size ranges from 0.025 to 0.25 micron. Once these solids become part of a giant molecular cloud, they can become coated in ices of water, CO, CO_2_, and ammonia that can be processed by UV photons generated by galactic cosmic rays [3,4]. Processing produces more complex molecular ices as well as an organic coating on the grains. These materials are heated as they fall into the solar nebula.

In order to gauge the degree of heating and the severity of processing of these grains in the hot inner solar nebula, we will compare the initial theoretical distribution described above with the matrices of carbonaceous chondrites. Silicate matrix grains range from ~5 microns down to the nanometer-scale. Other components of primitive meteorites are much larger (mm–cm), while carbon is generally <<10% by mass. This implies that virtually all (>99%) of the presolar silicate grains were at least melted (if not vaporized) in order to transform them into this much larger size distribution [5]. If nearly all silicates were processed at temperatures sufficient to melt and fuse grains into larger size aggregates, then the accompanying carbonaceous materials must have reached similar temperatures and been processed through related reactions.

The initial solid-state chemical distribution contained ~8 × 10^6^ carbon atoms (e.g., 88% of carbon in graphite grains) for every 1 × 10^6^ silicon atoms, not counting any carbon added as an organic coating on grain surfaces. In order to calculate the oxygen abundance within the grains, we take the Si + Mg + Fe cosmic abundance of 2.974 × 10^6^ atoms and assume that the initial valence state of Si is (+4), Mg is (+2), and Fe is (half +2 and half +3). If all metals are saturated oxides, then the amount of oxygen in dust is ~4.087 × 10^6^ atoms, again not counting any oxygen in organic dust mantles. If this were a closed chemical system, then all of the silicon, magnesium, and iron would be reduced to the respective metals, roughly half of the carbon would be converted to CO, and half would remain as graphite.

Since most metals in meteorite matrices are oxidized and carbon is an insignificant fraction of their total mass, thermal processing must have occurred in an open system. The cosmic abundance of nebular oxygen is ~18.8 × 10^6^ for every 10^6^ silicon atoms, so oxygen is roughly twice as abundant as carbon and occurs primarily as CO, O, OH, and H_2_O at high temperatures. There is sufficient nebular oxygen to oxidize all of the remaining carbon to CO and CO_2_, and all of the metals back to oxides (if any were actually reduced in the first place), and still leave ~10^7^ oxygen atoms in water and other gas phase oxides.

Connolly et al. [6] have demonstrated that the addition of a small fraction of carbon (as graphite or diamond) to an experimental charge of olivine and pyroxene grains produces excellent chondrule analogs. In addition, during rapid experimental heating, the internal redox state of the melt does not immediately equilibrate with the gas mixture used to control the oxygen fugacity of the experiment. Instead, the microenvironment inside the experimental charges is much more reducing than the gas and therefore, produces metallic grains within the melt rather than just along the surface of the charge. These chondrule analogs closely resemble natural samples.

In summary, the processing of the silicate grain population from its presolar size distribution into much larger planets, asteroids, calcium aluminum inclusions (CAIs), chondrules, and matrix implies that the associated carbonaceous materials were exposed to similar temperatures in a sufficient excess of oxygen to convert all carbon atoms into CO and CO_2_. While some molecular-cloud carbonaceous dust did survive processing in the nebula [7], much in the same manner as did some presolar oxide grains [8], such materials constitute only a small fraction of meteoritic carbon and are clearly distinguished by elevated D/H, ^15^N/^14^N, ^13^C/^12^C, or other isotopic ratios or noble gas patterns. Under such circumstances, why does carbon exist beyond trace amounts in the inner solar system at all?

## 2. Where is the CO in Protostars?

If we base estimates of the amount of CO expected to be in the gas phase in the cores of protostars on cosmic abundances and assume that virtually all carbonaceous dust and grain coatings falling into these systems reacts to form CO, then the CO to hydrogen ratio should be roughly 6.6 × 10^−4^. This is larger than the canonical value of 1 × 10^−4^ assumed when converting observations of CO in astronomical objects to mass. More realistic nebular models would predict time and spatially variable abundances that should be testable using newer generations of high spatial resolution telescopes. Such instruments will be capable of observing the effects of a large scale carbon cycle in protostars that repeatedly destroy solid carbonaceous grains and grain coatings in the hot inner nebula, converting them to CO, in competition with reactions on metal and silicate grain surfaces that act to convert CO and hydrogen into complex carbonaceous materials.

Fischer–Tropsch-type (FTT) reactions have long been invoked as a source of meteoritic carbon [9,10]. More recent studies have demonstrated not only that a wide range of solid surfaces support FTT reactions, but that the carbonaceous material deposited onto the grain surfaces is often a better catalyst than is the original surface [11]. There were many materials that we could have tested in addition to pure iron (the typical FTT catalyst) that are abundant in protostellar nebulae, as represented in the unequilibrated matrix of carbonaceous chondrites. We chose iron metal, iron oxide (magnetite), and iron silicate to test a range of iron-containing species. Each had a different reaction efficiency, different temperature dependence, and slightly different products, but all converted CO back into both solid and gas phase species at different ratios [11]. Because these reactions produce a wide range of carbonaceous solids and gases and are catalyzed by many different materials, they are not strictly speaking FTT reactions. To avoid confusion, we will refer to such catalytic reactions as Surface-Mediated Reactions (SMRs). SMRs can occur at temperatures as high as 900 K, though at such high temperatures, the organic material is quickly converted to “inorganic” carbon, such as carbon nanotubes [12]. At low temperatures (e.g., 200 K), reaction rates are very slow, although one predicts the production of smaller and more volatile hydrocarbons. At intermediate temperatures (~500–800 K), reaction rates are high enough to produce both volatile organics as well as an active macromolecular carbonaceous catalyst on the grain surface to promote continued conversion of CO and hydrogen into more complex hydrocarbons. These higher temperatures occur closer to the protostar, where nebular pressures are also higher, thus maximizing grain surface interactions and SMR reaction rates.

Protostars are ideal environments for SMR reactions: hydrogen and dust surfaces are both plentiful. The rate controlling step in the synthesis of carbonaceous materials is the initial adsorption of CO onto the grain surface [13]. Whereas earlier studies of the FTT reaction assumed that iron grains were required for this step, it is now understood that any solid particle can serve this purpose, greatly expanding the useful catalytic surface area in a protostellar nebula [11]. Once adsorbed onto the grain, surface interactions weaken the CO bond sufficiently that it can react with the abundant hydrogen colliding with the active surface. The result of such reactions is the formation of a variety of carbonaceous materials, including volatile organic molecules [10] as well as a macromolecular, high-molecular-weight, organic grain coating. While it was originally assumed that the formation of such a coating would cover the active catalytic sites on grain surfaces, shutting down FTT reactions, we now know that the opposite situation occurs for most dust surfaces, e.g., these coated surfaces become much more effective catalysts than were the original grains [11]. We therefore have a situation in most protostellar nebulae where CO production will lead to faster rates of reaction on grain surfaces, causing increasing catalytic activity on those surfaces and the reduction in CO in the gas.

As material falls towards the central star, both the pressure and average temperature increase, thus increasing the rate of SMRs in the innermost nebular regions. Increased temperature and density also lead to increased turbulence, lightning, and shocks that generate CO both from the initial infalling ISM carbon as well as from newly synthesized SMR products. Increased CO concentrations lead to faster SMR reaction rates, since the rate limiting step is the adsorption of CO onto active grain surfaces. These trends lead to a kinetically controlled system where the CO abundance as a function of radius from the protostar will depend on a wide range of parameters including grain composition and surface area, time since the most recent shock or lightning bolt, as well as the temperature and pressure. Of course, eventually, the thermal regime close to the protostar will dominate everything and any remaining solid carbon will be converted to CO: the radius at which this occurs will depend on the mass of the central star, the rate of mass accretion, and the age of the nebula.

If all protostars transform the soot-like carbonaceous interstellar dust into CO, then there should be an observable increase in CO within the hot central cores of such stellar nebulae. In these hot inner regions, the ratio of CO to hydrogen should increase by up to a factor of six from the canonical value of 1 × 10^−4^, due to reaction of the carbonaceous grains with the abundant—and oxygen-rich—amorphous silicates. CO generated too close to the central star could be lost via bi-polar outflows. Similarly, carbonaceous grains formed at such high temperatures on grain surfaces could be quickly transformed into carbon nanotubes [12] and could also be lost to the system via bipolar outflows. CO generated near the mid-plane will be convected to cooler areas [14], where it can begin SMRs to form organic molecules and carbonaceous solids. We therefore predict that there will be a sharp peak in CO within about 1A.U. of a central protostar and that an elevated level of CO/H_2_ could extend out to about 3–4 A.U., brought about by distributed CO production in nebular lightning and shocks and possibly, by production in protoplanetary sized bodies disrupted by energetic collisions that is partially balanced by the reaction of CO via SMRs. This region will also produce a rich array of simple organic molecules via SMR. The size of the CO production zone will depend on the mass and age of the protostar with more production around larger, younger stars.

Outside about 5 A.U., production of carbonaceous dust and organic molecules should be minimal. However, with time, the transport of volatile organics produced in the inner nebula should gradually increase the levels of species such as methane, ethane, propane, and other straight chain hydrocarbons together with nitrogen-bearing species such as methyl cyanide, methyl amine, or ammonia (produced by the analogous Haber–Bosch reaction of N_2_ and H_2_ on grain surfaces), as well as more complex organics such as acetone, toluene, ethyl benzene, or benzoic acid [10]. If we could follow the chemical evolution of a single protostar as a function of time, we would first see the rapid increase in CO/H_2_ in the central nebula, followed by a gradual decrease in CO as the rate of SMRs begins to utilize the abundant CO for the production of carbonaceous solids and organic molecules. The abundances of such molecules will begin to build up in the outer regions of the nebula as organics produced in the hot dense interior regions of the nebula are transported outward to at least 40–50 A. U. and beyond, where such volatile species can be incorporated into nascent cometesimals. This is similar to the gradual increase in crystalline silicate minerals predicted for cometesimals in the solar nebula [15].

## 3. Laboratory Studies of Surface-Mediated Reactions

**Figure 1:**
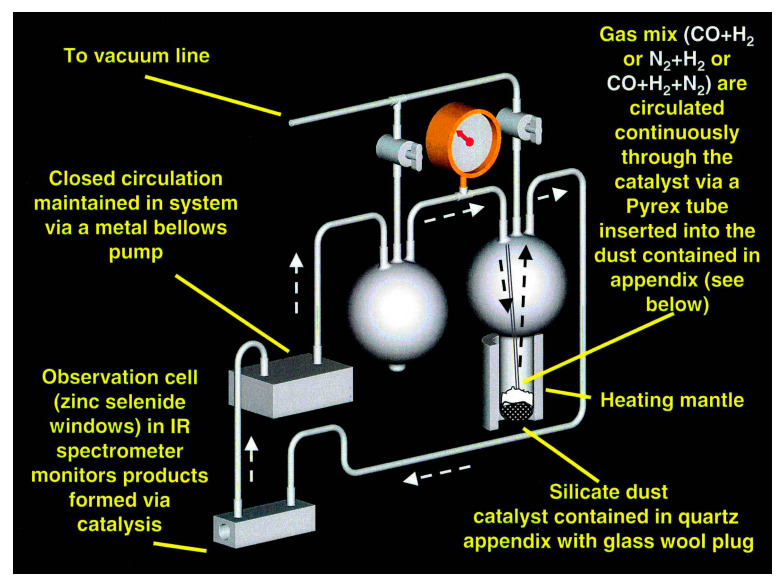
Three-dimensional drawing of the experimental system used for catalytic experiments. The catalyst is contained in the bottom of a quartz finger (attached to a 2-L Pyrex bulb) that can be heated to a controlled temperature. A Pyrex tube brings reactive gas to the bottom of the finger. The gas passes through the catalyst into the upper reservoir of the bulb and flows through a stainless-steel tube at room temperature to a glass-walled observation cell (ZnSe windows) in an FTIR spectrometer. A closed-cycle metal bellows pump returns the sample via a second 2-L bulb and the Pyrex tube to the bottom of the catalyst finger to start the cycle over again. We have ten identical experimental systems: the total volume of each system is 4.7 +/− 0.1 L.

The experimental system has been described previously [10] and is shown schematically in Figure 1. For these experiments, we always used the same gas mixture: 75 torr CO, 75 torr N_2_, and 550 torr H_2_ measured at room temperature, for a total initial gas pressure of 700 torr. The gas pressure increases as we heat the catalyst to our working temperature, then changes as the CO and H_2_ are converted to various products during the course of the reaction. We note that we do not heat the tubing used to circulate the gas in this system, so that in many of the experiments, the pressure of water generated via the reactions in the system exceeds the equilibrium vapor pressure of water at room temperature, and measurements of the concentration of water in the system, therefore, reach a plateau at just under 20 torr. Condensation of the excess water vapor throughout the experimental system depletes the initial oxygen in the system and, therefore, could suppress the formation of CO_2_. The FTIR spectrometer records the spectrum of the infrared active gases in the system at regular intervals, depending on the rate of change we expect for a particular experiment, and is set to provide a reasonably continuous record of the CO depletion and CH_4_ generation as a function of reaction time for a given experiment.

We are aware that the pressures used in our experiments are much higher than those in the solar nebula and are also aware that this difference could affect the results of our experiments. We would love to duplicate nebular conditions and run our experiments continuously at nebular pressures for several hundred years, but such timescales are impractical. On average, our experiments run for from several days to several weeks. If an average experiment lasts for a week (6.05 × 10^5^ s) but should last for a century (3 × 10^9^ s), to duplicate conditions found in protostellar nebulae, we can obtain the same number of reactive CO collisions with our surfaces by increasing the pressure by a factor of ~5 × 10^3^, from about 10^−4^ to 10^−3^ atmosphere in the nebula to between 0.5 and 5 atmosphere in our experiments. The experiments that we run at about one atmosphere, therefore, underestimate the number of collisions experienced by an average grain surface in the nebula in 100 years. Grains could circulate in such nebulae for 10^4^ years or even longer (but at differing temperatures). We consider pressure to be a proxy for time and will generally ignore its effects on our products in these experiments. We have used this apparatus to carry out a variety of experiments to measure the characteristics of SMRs.

### 3.1. Carbon Isotopic Fractionation

One set of experiments was designed to measure any possible fractionation in carbon isotopes as a function of temperature using an Isotope Ratio Mass Spectrometer. While we did find that carbon isotopes became lighter as temperatures increased [16], the magnitude of the change was relatively trivial. Compared to the initial CO gas composition, the δ^13^C value of the solids decreased from −42 per mil at reaction temperatures of ~575K to −50 per mil for solids synthesized at ~875 K, with average δ^13^C measurement uncertainties of +/−1.2 per mil. It is unlikely that any carbonaceous grain will be formed exclusively at a single temperature in the nebula. It is much more likely to form at a variety of temperatures as turbulent convection and gas drag circulate the grains above and below the nebular plane as well as both towards and away from the Sun. Even small excursions within the nebula could result in significant changes in temperature and overprinting of the isotopic signature. This null result was published [16] as a warning to those who might otherwise attempt to use carbon isotopic measurements to infer the formation temperatures of carbonaceous materials in samples from primitive asteroids, comets, and from meteorites.

### 3.2. Carbon Gas to Solid Branching Ratios

Quantitative measurements of all gases in the system are made by generating synthetic spectra using the High-Resolution Transmission (HITRAN) spectroscopic database [17] and comparing these data with experimentally measured FTIR spectra [18]. These gas species include CO, CO_2_, CH_4_, C_2_H_6_, C_2_H_2_, NH_3_, and H_2_O. CO is the only source of carbon in the reaction system. Because we do not have a method to directly measure the number of moles of carbon deposited onto the catalyst surface, we measure this quantity indirectly by subtracting the molar quantities of CO, CO_2_, CH_4_, C_2_H_2_, and C_2_H_6_ in each spectrum from the initial number of moles of CO in the system and we assume that the difference was deposited onto the surface of the catalyst. Since CO_2_, CH_4_, C_2_H_6_, and C_2_H_2_ constitute the overwhelming majority of the carbonaceous gas phase products observed in our system, and the quantitative calculation of the gas phase concentrations from FTIR spectra are accurate to at least 10% or better, we believe that this assumption is reasonable. The fraction of the initial carbon available in the original carbon monoxide feedstock deposited on the grain surface can be calculated from this information.

**Figure 2:**
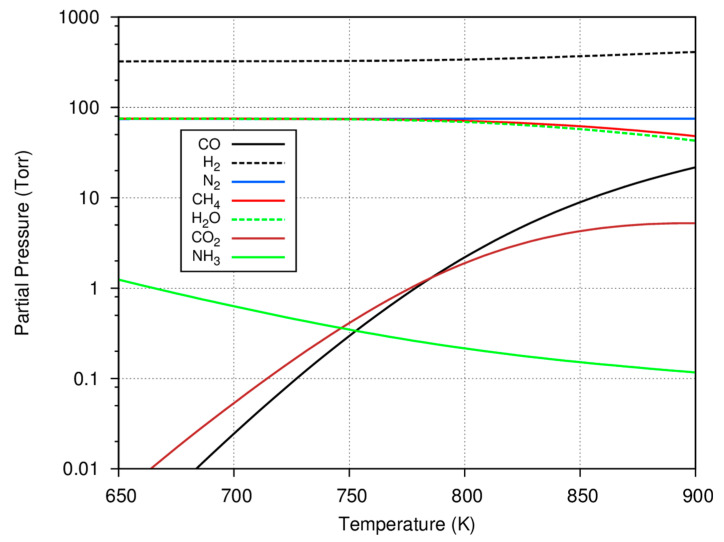
The equilibrium partial pressures as a function of temperature for an initial mixture consisting of 75 torr of CO, 75 torr of N_2_, and 550 torr of H_2_ calculated based on the Fischer–Tropsch, Haber–Bosch, Boudouard, and Water–Gas Shift reactions.

Before considering the experimental data, some insight can be gained by further considering what species are predicted to be present in an equilibrium mixture based on a typical initial gas charge. This information may be useful in assessing catalyst selectivity to the methanation reaction or the formation of deposited carbon and hydrocarbons. To compute the equilibrium gaseous concentrations with such multiple reactions, the minimization of Gibbs free energy technique using Lagrange multipliers was used [19] with a starting gas mixture of 75 Torr carbon monoxide, 75 Torr nitrogen, and 550 Torr hydrogen. The results of these calculations for the major reactant and product gases are shown as a function of temperature in Figure 2.

As shown in Figure 2, the formation of methane is favored at lower temperatures, with virtually all of the carbon monoxide consumed and converted to methane. As the temperature increases, the reaction shifts, less methane is predicted to be formed, and there is residual carbon monoxide as well as some carbon dioxide in the equilibrium mixture. Since the consumption of carbon monoxide is used to determine the progress of the experiments, it is important to note that not all of the carbon monoxide may be consumed at the higher system temperatures. Because of the low system pressure (compared to those used in the industrial Haber–Bosch process), only a small amount of ammonia is predicted to be formed and larger, equilibrium amounts of ammonia are also favored at the lower temperatures. In several experiments, it is common to form ~50–60 Torr of methane; in these cases, it is clear that the overall reaction at equilibrium favors the methanation reaction and the selectivity to form condensed hydrocarbons is low.

**Figure 3:**
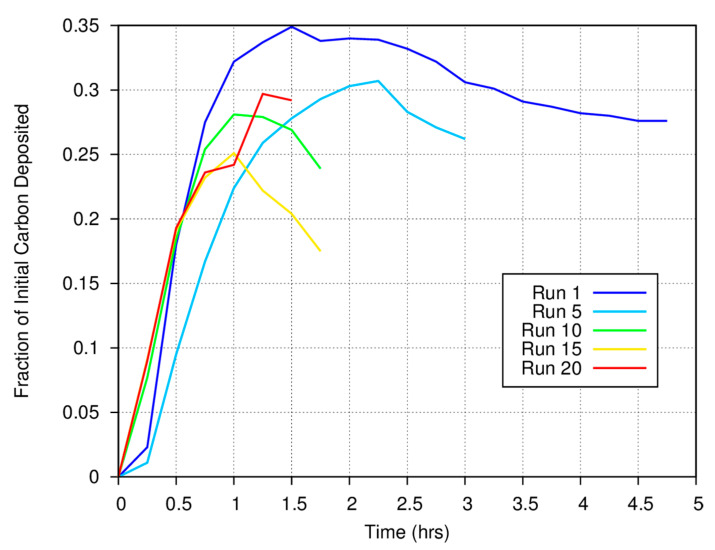
The fraction of the available carbon from CO that is deposited onto a magnetite catalyst at 773 K as a function of time during various experimental runs. Earlier runs generally deposit a larger fraction of the available carbon as solids. Note the anomalous “kink” at 0.25 h in runs 1 and 5.

Figure 3 shows the measured carbon gas/solid fraction for a magnetite catalyst as a function of time and run number for a series of experiments conducted at 773 K. Approximately 25% of the carbon in the initial CO is deposited onto the surface of the Fe_3_O_4_ catalyst by the end of each experimental run, while the bulk of the carbon remains as gas phase CO, CO_2_, CH_4_, and C_2_H_6_. Note the anomalous behavior at the start of runs 1 and 5. We always measure the initial CO concentration and perform quantitative measurements using the synthetic spectra generated using the HITRAN database at the temperature of the FTIR cell (room temperature) and the measured total system pressure. The anomaly results because there is a rapid rise in total system pressure when heat is applied to the catalytic “finger”, thus increasing the concentration of gas phase species in the FTIR cell.

For the first measurement made at the start of a run, the pressure will be 700 torr (by design at room temperature). However, because the catalyst finger is now being heated to 773 K (for the data shown in Figure 3) as we start the system, the temperature of the finger will be somewhat higher, thus increasing the pressure throughout the closed system. A higher pressure in the system will lead to an artificial increase in the gas phase abundance of carbon as determined from comparison between synthetic and measured spectra for the second and all subsequent measurements until the surface-mediated reactions begin to deposit carbon onto the grains or until fewer moles of gas phase species are formed as reaction products. If carbon deposition or the overall reaction rates are slow, the negative anomaly will persist for some time. For faster rates of deposition, carbon will be taken out of the gas phase more rapidly and increasing gas pressure can be compensated for by carbon deposition. Note that in Figure 3, the anomaly is pronounced for run 1, less pronounced in run 5, and much less obvious in runs 10, 15, and 20. As the catalytic finger was heated at the same rate for each experimental run, these data imply either that the rate at which carbon is deposited onto the surface of the catalyst at the beginning of each experiment increased as a function of run number or that the overall reaction rate increased. Thus, the more carbon that has already been deposited onto the magnetite surface, the faster carbon is deposited onto that surface in subsequent experiments.

**Figure 4:**
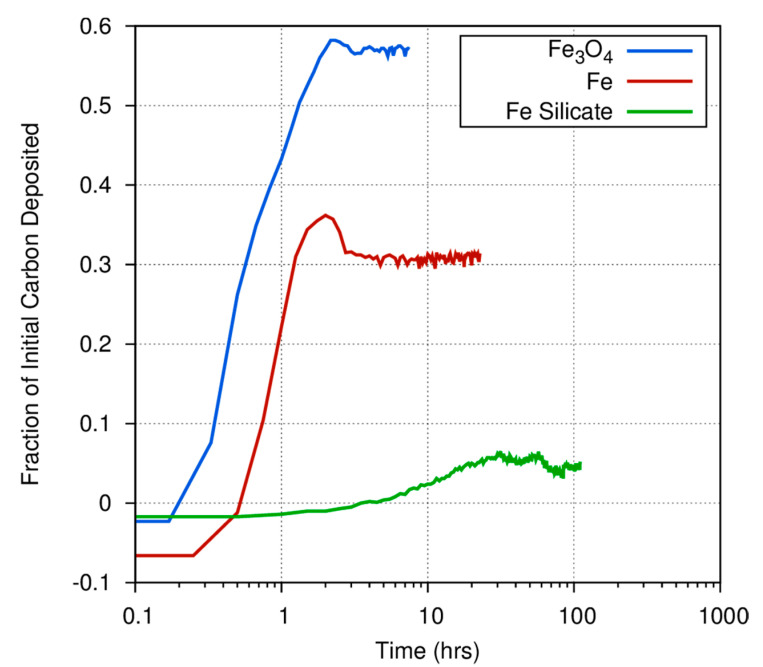
The fraction of the available carbon from CO deposited onto a magnetite, iron, or iron silicate catalyst during the initial experimental run at 873 K as a function of time shows a very wide range in gas/solid branching ratio.

**Figure 5:**
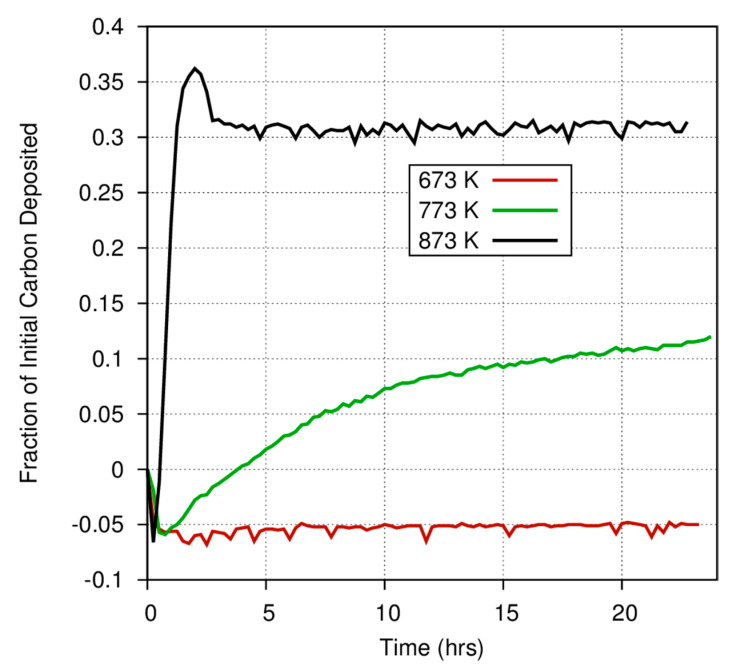
The fraction of the carbon available from CO deposited onto an iron catalyst as a function of time for several different temperatures during the first experimental run with a fresh catalyst.

Figure 4 shows the different initial rate and gas/solid fraction for carbon deposition onto iron, magnetite, and amorphous iron silicate smokes at 873 K. Fe_3_O_4_ very efficiently converts the initial C in the CO feed stock into a carbonaceous deposit, as compared to pure Fe or the amorphous silicate smokes. Figure 4 also demonstrates the considerable differences among catalysts in the fraction of carbon deposited onto grain surfaces versus the fraction that is converted into gas phase products. At 873 K, nearly 60% of the CO exposed to a magnetite catalyst is deposited onto the surface, whereas only 30% of the carbon exposed to an iron catalyst is converted into a carbonaceous deposit. Even at high temperatures, where the Boudouard reaction should become much more important, much less CO is converted into a solid on the amorphous iron silicate catalysts. The effect of temperature is more dramatically illustrated in Figure 5, where the fraction of CO deposited onto an iron catalyst surface at three different temperatures varies from 0.3 at 873 K to approximately 0.12 (accounting for the anomaly discussed above) at 773 K, and down to ~0.05 at 673 K. Obviously, for a pure Fe catalyst, the branching ratio of the carbon deposited onto the grain surface compared to the carbon incorporated into volatile compounds is a very strong function of temperature. Higher temperature yields fewer volatiles. This is consistent with the general trend in the Boudouard reaction but is not consistent with thermodynamic predictions of the instability of free carbon in the presence of excess hydrogen. More information on these experiments is available in Nuth et al. [20].

### 3.3. Surface Area and Morphology of the Carbonaceous Solids from SMRs

Surface-mediated reactions can occur at active catalytic sites on almost any grain surface [9,10] and we have previously demonstrated that the deposition of refractory carbonaceous material on grain surfaces can actually enhance the rate of such reactions [11]. We had made the simple assumption that the carbonaceous coating grew to cover the surface of the underlying catalyst, leaving an outer coating that completely and uniformly covered the original grain. Such “grain varnishes” or refractory organic residues have frequently been described in the past as the result of ultraviolet or radiation processing of ices containing simple organic precursors such as CH_4_, CH_3_OH, CH_2_O, and NH_3_ [21,22]. It seemed natural to assume that a similar morphology would result from surface-mediated processes. That assumption is incorrect.

We used a Quantichrome Nova E series surface area analyzer and the Brunauer–Emmett–Teller (BET) technique [23] with N_2_ as the adsorbed gas to measure the area of our initial pure magnetite catalyst, as well as small samples of the catalyst that had been used for 5, 10, 15, and 20 experimental runs. We found that the area of our catalyst increased significantly at each measurement. This implies that the area increases during each individual run as well, making quantification of the reaction kinetics as a function of the catalytic surface area an interesting challenge that we will tackle in future work. The results of these measurements for an initially pure magnetite catalyst are given in Table 1.

As can be seen, the active catalytic surface area more than doubled relative to the surface area of the original magnetite catalyst within the first five experimental runs, then increased to more than four times the initial magnetite surface area after 10 runs and to more than six times the original area after 20 experiments. It is highly unlikely that the catalyst remains pure magnetite as its surface area increases.

Unfortunately, these results should be taken as lower limits to the actual increase in surface area that occurred during these experiments. As we began to quantitatively measure the reaction rates per unit surface area of catalyst, we became aware that the rates did not scale by the area of the catalyst as we had expected. Instead, the rate for iron powders compared to the rate for several lengths of iron wires indicated that most of the iron powder was not seen by the reactive gas mixture. The experimental system shown in Figure 1 was set up as a fluidized bed reactor, in which the gas injected at the bottom of the catalyst bed “liquefies” the very fluffy iron silicate smokes that we first tested as catalysts. In such a system, the gas interacts freely with the entire catalyst bed. Use of the much denser iron, hematite, or magnetite powders in this system eliminates the fluidity of the catalyst at the gas flow rates available in our system and even though we thought that the gas permeated the entire reaction bed, it became obvious to us that a significant portion of the iron powder catalyst did not participate in the reaction. While this does not change the product distribution of the reactants, it certainly effects measurements of the reaction kinetics and means that measurements of the surface area per gram of an iron or magnetite powder catalyst, measured for a randomly drawn sample of the bulk material, averaged the increased surface area of active grains with a larger quantity of grains that had minimal interaction with the gas.

Even this lower limit for the rapid increase in the surface area of the catalyst was very difficult to explain based on our previous experiments and with our ingrained assumptions concerning the morphology of the deposited coating. Previous analyses of the coating on iron silicate smokes with initial surface area ~125 m^2^/g and a total mass less than 5 g showed that after 20 runs, the grains were 10% by mass carbon and 0.2% by mass nitrogen [24]. Increasing the surface area of our catalyst by a factor of six would require more than doubling the average radius of the 325 mesh magnetite catalyst and would require much more than the available quantity of carbon in the system to accomplish this increase. Based on our previous work, we would expect that much less than a gram of carbon deposition occurs (a total on all grains in the system) over 20 runs and we know that a significant fraction of the available carbon is converted into CO_2_, CH_4_, and other volatile organic species via the overall reaction [11]. This does not leave enough mass to coat the initial magnetite grains to sufficient thickness to increase their surface area by factors of four or more. Continuous carbonaceous coatings on the catalytic surfaces can therefore be ruled out by these measurements—a more complex surface morphology is required.

**Figure 6:**
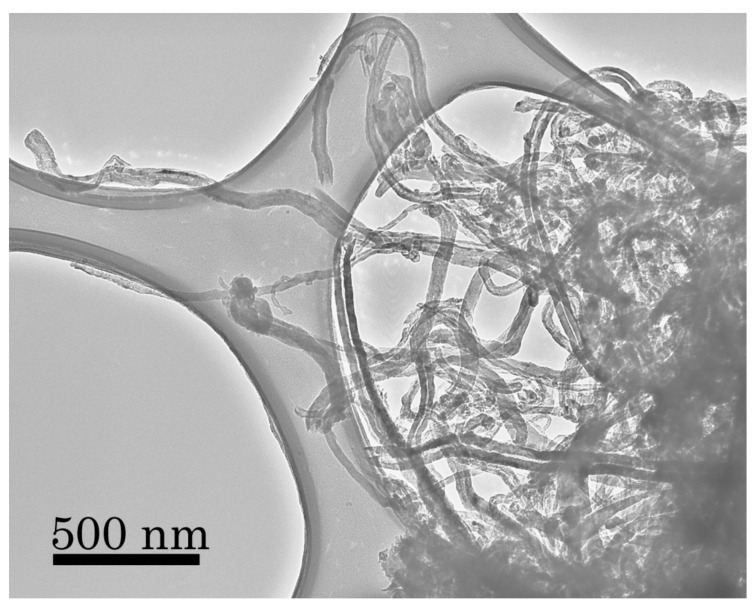
Surface-mediated reactions produce very high surface to volume deposits on graphite grains used as catalysts, as can be seen in this TEM image of carbon nanotubes grown from our standard gas mixture (75 torr CO, 75 torr N_2_, 550 torr H_2_) at 873 K [20]. We had previously believed that such extensive nanotube growth was unique to graphitic catalysts and was due to the ready supply of carbon atoms available from the graphite grains themselves. The conditions of SEM observation are as follows: the accelerating voltage is 30 kV, the pressure is 0.1 mtorr, the sample is sputter coated with Au-Pd at an emission current of 100 mA.

In previous experiments conducted at 873 K using a graphite catalyst, we have shown the formation and growth of carbon nanotubes [12], essentially a thin string of carbon with a very high surface area per unit mass. A TEM image of these deposits is shown in Figure 6 to illustrate the very large surface area possible for such morphologies that we had believed were unique to experiments using graphitic catalysts and their large reservoir of potentially reactive carbon. We had never seen evidence for nanotube formation at temperatures less than 823 K for any other catalyst, including in both SEM analyses as well as in electron diffraction analyses of lower temperature grain coatings. In our previous SEM images, the surface always appeared to be “lumpy”—which we attributed to grain clumping and other macroscopic features produced during the reaction.

**Figure 7:**
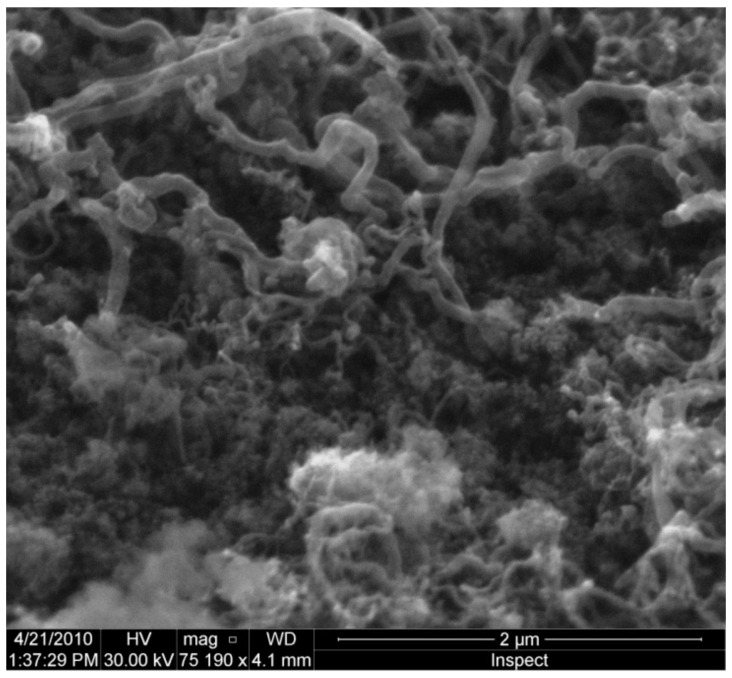
SEM secondary electron image of an iron-silicate sample after sixteen heating cycles at 873 K. The conditions of SEM observation are as follows: the accelerating voltage is 30 kV, the pressure is 0.1 mtorr, the sample is sputter coated with Au-Pd at an emission current of 100 mA. CO is the only source of carbon available for the growth of nanotubes in these experiments [25].

Higher resolution SEM images of iron silicate smokes used as catalysts at 873 K tell a very different story, as can be seen in Figure 7. It appears that active regions on the surface of the catalyst initially promote carbon deposition in the local area. However, rather than spreading uniformly over the surface of the grains, the carbon deposit also begins to grow away from the catalyst surface. While, in some ways, this initially appears to resemble VLS (Vapor–Liquid–Solid) growth along a crystalline “c-axis”, the resultant deposit is neither crystalline nor is it a smooth needle or whisker—in fact, it is rather clumpy and quite irregular. It also appears that much, if not all, of the deposited carbon is capable of promoting additional surface-mediated reactions and further carbon deposition much as we had observed when using the graphite catalyst. The newly deposited carbon does not appear to thicken the nanotubes themselves, but instead, appears to cause them to lengthen. Based on experiments on the formation and growth of nanotubes [12], we know that carbon atoms are relatively easily mobilized from such initial condensates to form longer nanotubes—another similarity to VLS growth. It is possible that once such tubes begin to form, the chemical composition of the original catalyst becomes increasingly less important with time.

The formation of filamentous carbon deposits is one possibility that may explain both the dramatic increase in specific surface area and the increased catalytic activity. Such filamentous carbon is known to occur in carburizing and reducing atmospheres in the 400–800 °C range. Such deposits have been known for some time; one of the first reports of such deposits was made after observations of the reaction of carbon monoxide with iron oxide on the brickwork in a blast furnace [26]. Schulz et al. [27] found such structures while studying Fischer–Tropsch and methanation catalysts and termed the deposits “carbon expanded iron”. It was suggested that, under certain conditions, the carbon deposition broke the bulk metal into finer particles [27]. The carbon would grow into filaments that would keep the metal particles from agglomerating, thereby making the metal more accessible to reactant gases. Schulz et al. [26] noted the unexpected catalytic activity per gram of material after the growth of these carbon-expanded iron filaments. Such behavior may be responsible for the increased catalytic activity noted with the current catalysts, where reaction rates increase with successive runs. However, in our initial experiments (e.g., Figure 7), the underlying catalytic dust is amorphous iron silicate rather than iron metal.

In industrial processes, such carbonaceous deposits can occur on the steel reactor walls, causing harmful effects. One particular growth of carbon, called “metal dusting”, causes the corrosion of vessel walls, resulting in a dust of graphite and small metal particles [28]. Both the filamentous carbon and metal dusting growth occur with metals that absorb carbon, e.g., iron, nickel, and cobalt. Carbon is absorbed and transported within the metal due to a gradient in carbon activity. In fact, the diffusion of carbon in the catalyst particle is thought to be the rate-determining step in the reaction sequence because there is very close agreement between activation energies for filament growth and those for diffusion of carbon through the metals [29].

The formation of filamentous carbon deposits on metal particles is outlined in six steps given by Bonnet et al. [30]. These steps include: (1) the decomposition of hydrocarbons on the metal surface and supersaturation of the metal with absorbed carbon; (2) the nucleation and growth of cementite, Fe_3_C; (3) carbon diffusion becomes impeded through the cementite layer and hinders further transfer from the gas phase; (4) graphite begins to precipitate on the cementite surface, reducing the activity of carbon at the graphite/cementite interface and thus, making cementite unstable and causing it to decompose into carbon and iron; (5) these free carbon atoms attach to the basal planes of graphite that grow into the cementite while the iron atoms agglomerate to form small particles; and finally, (6) these small particles are able to act as catalysts for further carbon deposition and growth. While these steps outline the growth of filamentous carbon with metallic iron, Bonnet et al. proposed a new mechanism whereby iron oxides can also form such filamentous structures by being converted directly to cementite without having metallic iron as an intermediate phase [30]. This filamentous carbon should thus be able to form with iron oxide mixtures as well as on metallic iron covered with oxide layers. Thus far, the experimental evidence seems to support the formation of this filamentous carbon and further experiments will be focused on imaging and chemical analyses of such deposits using high resolution microscopy to investigate this possibility.

### 3.4. Implications of Filamentous Carbon Solids for Nebular Processes

Our experiments indicate that macroscopic carbon particles, many hundreds—or even thousands—of nanometers in length and quite irregular in shape, could form at least on the surfaces of amorphous iron and magnesium silicates, iron, magnetite and graphite grains, and probably on many other surfaces as well. This could have several consequences. First, such carbon-rich growth could at least partially replenish some of the presolar, graphite-like dust previously destroyed in high temperature nebular shocks, lightning plasma tubes, or in grain aggregates heated while in close proximity to the early sun. While we know that a large fraction of presolar silicate grains were processed in the nebula to produce the much larger dust particles found in meteorite matrix, presolar carbon dust must have experienced similar heating processes, though no quantitative measure of such processing is yet available in the literature. However, while silicates are converted into liquid droplets or vapor at high temperatures—and silicate vapor can recondense—carbon is converted into CO or CO_2_ at high temperatures in the oxygen-rich solar nebula or when in direct contact with silicate grains. Surface-mediated reactions would be a significant pathway to convert such gases back into the solid carbon species found in meteorites.

**Figure 8:**
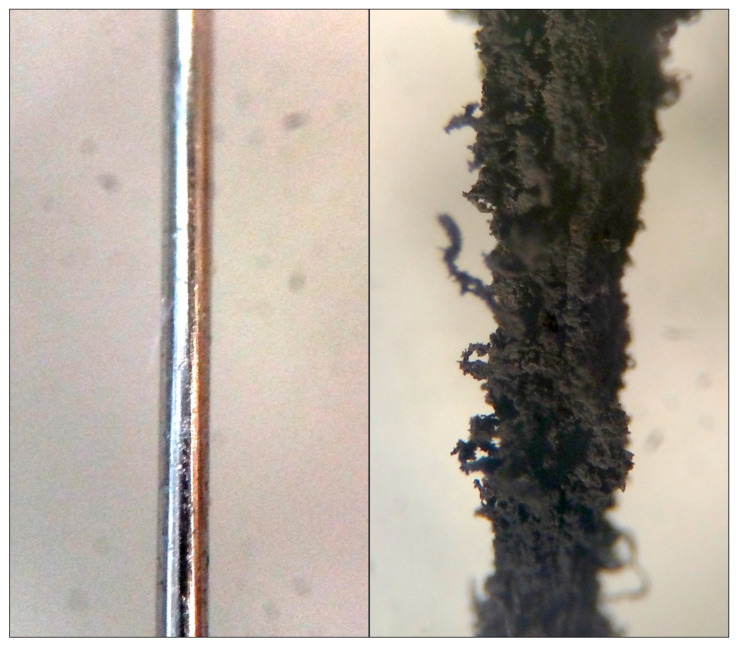
A microscope image of a section of iron wire used in the experiments (0.009 inches in diameter) shown at the same scale before (left) and after (right) it served as the catalyst for a single experimental run of CO + N_2_ + H_2_ ≥ products at 873 K [20].

An interesting possibility, if such processes occur on the surfaces of larger (mm-scale) silicate and metallic grains in the nebula, is that such growth could act much in the same manner as do fresh snowy condensates on the surfaces of the larger ice grains in Saturn’s rings. An example of this is shown in Figure 8, where a section of iron wire is compared before and after use as a catalyst for a single experimental run [20]. Such fluffy grain coatings could damp out the momentum in gently colliding particles, changing the coefficient of restitution and promoting grain–grain sticking [31,32]. Surface-mediated reactions would happen fastest in the higher temperature, higher pressure regions of the inner solar nebula, just where chondrules, CAIs, and other macroscopic meteoritic components are also forming. Under such circumstances, the solid carbonaceous products would tend to be more graphitic and could even be similar to the stacked-cup nanotubes that we have observed previously [12]. If some of these refractory surfaces served as catalytic sites for the growth of filamentous “whiskers”, then such surface growth could also promote the aggregation of these larger meteoritic components. In fact, at least one study has reported an association between carbon nanotubes and CAIs [33]. Such carbonaceous growth could have been the “Velcro” that promoted the aggregation of meteorite parent bodies in the high temperature inner nebula.

Finally, we have previously suggested that “Fischer–Tropsch-Type” surface-mediated reactions could convert CO or CO_2_ produced in high temperature nebular events back into carbonaceous gases and solids [34]. These processes would have two major consequences. First, the conversion of CO into more reactive carbonaceous species breaks down the assumed stable C^16^O reservoir that is required for the CO self-shielding mechanism [35,36,37,38] to enrich the solar nebula in heavier oxygen isotopes and produce mass independent oxygen isotopic fractionation in such meteoritic components as CAIs and chondrules [39,40]. Second, solid carbon fibers are much more easily incorporated into growing planetesimals in the warm inner solar nebula than would be the host of gaseous hydrocarbons generated by SMRs [10]. These carbonaceous solids could serve as a reactive feedstock to synthesize a wide range of organic biomolecules as they react in the presence of water and amorphous magnesium and iron silicates in evolving planetesimals. We are not claiming here that SMR reaction products constitute the single—or even the most important—mechanism that forms the full distribution of organic molecules in asteroids or comets or that are found in meteorites or interplanetary dust particles. There are many different processes that can be important sources for particular compounds in many different meteorite types [41]. However, we do believe that surface-mediated reactions are a very efficient mechanism for converting nebular CO or CO_2_ into solid carbonaceous materials and the products of such reactions have not yet been investigated.

## 4. The Great Carbon Cycle Operating in the Inner Solar Nebula

As discussed above, interstellar grains are an intimate aggregate of carbonaceous and silicate dust. In the giant molecular cloud that preceded the collapse of the solar nebula, these grains and aggregates may have been coated by an organic varnish via radiation processing of icy, carbon-bearing mantles. As these presolar grains arrived in the innermost regions of the primitive solar nebula, they were subjected to high temperatures near the Sun as well as to high-temperature shocks, lightning, magnetic reconnection events, and other energetic processes that resulted in the melting or evaporation (and recondensation) of nearly 99% or more of the silicates in these aggregates. If we subject an intimate mixture of metal oxides and carbon to high temperatures in a reducing environment, the result is CO plus CO_2_ plus metal—we call this industrial process smelting. However, the solar nebula is a very oxidizing environment and the metal grains will be quickly oxidized by reaction with water vapor, O, OH, or even CO_2_, while the carbonaceous solids are simply destroyed, resulting in higher local concentrations of CO. We note however, that highly reducing, non-equilibrium microenvironments can be produced within carbon-rich, silicate aggregates during rapid heating events [6] and that the products of such reactions are very similar to natural chondrules.

As the shocked gases cooled below about 900K, the CO and H_2_ could begin reacting on surfaces of the recondensed silicates and metals, forming elongated filamentous carbon fibers rather than grain coatings. Because only the ends of the carbon filaments were in contact with the oxide grains, another high temperature shock would simply break this connective joint by reactive oxidation, leaving a free-floating carbon fiber and an oxide grain. Due to the nature of the surrounding gases (H_2_O, OH, and O atoms), some filaments could be partially or completely destroyed depending on the temperature, pressure, and duration of the shock. However, as the carbon grains are no longer nanometer-scale and intimately mixed with tiny oxides, the survival rate for these larger carbonaceous filaments should be higher than for the original presolar aggregates.

Since the rate limiting step of Fischer–Tropsch-type reactions is the adsorption of CO onto reactive grain sites [13] and SMRs are simply a much more generalized version of this idealized reaction type, the SMR reaction rate will increase in proportion to the CO concentration. Therefore, the more presolar carbon destroyed per unit volume in high temperature shocks, the faster filamentous carbon will be generated by SMRs due to higher concentrations of CO. In addition, as many of these carbon grains will be generated over a range of temperatures in cooling gas packets, carbon isotopic signatures of such grains will not be particularly distinctive. Finally, while SMRs do produce solid carbonaceous filaments, the major SMR reaction products are almost all volatile organic molecules such as alkanes, aromatics, and a wide range of more complex species for most of the natural catalysts and temperatures we have studied. Unlike the filaments which might act to increase the grain–grain sticking probability of inorganic dust, such volatile carbon molecules are not very likely to accrete into warm planetesimals in the inner solar nebula. However, if these freshly produced organics are transported into the outer solar nebula [15], they might contribute to the diversity of molecular species observed in comets formed later in nebular history.

The bulk C/Si ratio has been estimated for a variety of solar system bodies, starting with a presolar value of ~6 [42] (Table 1), based in part on previous work [43]. While the C/Si ratio of the Earth is depleted by ~3.5 orders of magnitude (C/Si ~ 0.001), comets (as exemplified by Comet Halley) show an enhanced C/Si ratio (~8), supporting possible transport of volatile, carbon-bearing species from the hot inner nebula where they were possibly synthesized via SMRs, to the much colder regions where comets formed. Carbonaceous meteorites show two order of magnitude levels of carbon depletion, while IDPs are only depleted by factors of ~3 as a class [44], consistent with an origin from both cometary and asteroidal sources. These data indicate that presolar carbon was very efficiently destroyed in the terrestrial planet region, but that SMR reactions acted to convert the higher concentrations of CO in this hot, dense region into both volatile organic molecules that could be transported outward as well as into solid carbon filaments that were incorporated into inner solar system planetesimals.

Presolar grain destruction in the inner solar system appears to have been extremely efficient, with more than 99% of silicate dust reworked into much larger, very different refractory constituents such as CAIs, chondrules, and left over grain matrix material that accreted into chondritic meteorite parent bodies. More than 99.95% of presolar carbonaceous materials were destroyed and lost from the inner solar system based on estimates of the C/Si ratios within the Earth, Moon, and chondritic meteorites. Since comets such as Halley appear to have an enhanced C/Si ratio, at least some of the presolar carbon must have been transported outward to comet forming regions and it is unlikely that this carbon traveled as highly volatile CO which would be less efficiently incorporated into growing cometesimals than would more complex and less volatile organics.

Data from the Stardust Mission [45] demonstrated the abundance of refractory inner solar system silicates such as CAIs and chondrule fragments within Comet Wild 2, a Jupiter Family Comet. However, since the cometary C/Si ratio of comets is enhanced relative to that of presolar materials, at least as much carbon, and probably many times more, must have also been transported from the hot inner nebula together with these grains. Given that the C/Si ratio in comets is enhanced to 8 compared to the presolar ratio (6) while 99.95% of the carbon in the inner solar system was converted to CO, on the order of at least a third of the CO generated in the inner nebula, and maybe all of it, must have been processed into more complex molecules and solids that could easily be incorporated into growing cometesimals. A major uncertainty in this estimate is the relative volumes of the hot, inner solar nebula compared to the volume where comets formed, in addition to the uncertainty in the relative masses of the two regions. Despite these uncertainties, the enhancement in the C/Si ratio of comets over that of presolar materials argues for significant production of complex organic molecules via SMRs from the CO feedstock generated via the destruction of presolar carbon in the inner nebula. It also argues for significant gas outflows from the inner to outer nebular regions in order to make a measurable enhancement in the C/Si ratio of the outer nebula, as evidenced by comets.

## 5. Summary

Presolar dust is an intimate mixture of tiny carbonaceous and silicate grains. More than 99% of silicate dust was processed at high temperatures to form the silicate components in meteorites. The associated processing of carbonaceous dust would produce large quantities of CO as 99.95% of the presolar carbon was destroyed. Much of the CO generated by the destruction of presolar carbon dust was processed on the surfaces of mineral grains via SMRs to produce carbonaceous molecules and fibers. While the carbonaceous fibers may have helped to aggregate silicate components into meteorite parent bodies, the volatile organic species were transported outward, along with some small refractory silicates, to enhance the organic content of comets such as Halley and Wild 2. The carbonaceous fibers in chondritic parent bodies could serve as a feedstock for the generation of more complex organic species during metamorphic processes on evolving asteroids.

## Figures and Tables

**Table 1:** Variation of catalyst surface area with run number.

Catalyst	Surface Area (m^2^/g)
Initial Magnetite Sample	7.41
Magnetite, 5 runs at 450 °C	19.65
Magnetite, 10 runs at 450 °C	33.95
Magnetite, 15 runs at 450 °C	35.31
Magnetite, 20 runs at 450 °C	49.46
